# CCDC6 Immunostaining in Conjunction with the Rad51 HRD Assay May Expand PARPi Treatment Eligibility in Patients with HGSOC

**DOI:** 10.1158/2767-9764.CRC-25-0455

**Published:** 2026-01-26

**Authors:** Daniela Criscuolo, Francesco Merolla, Benedetta Pellegrino, Luca Russolillo, Ilaria De Benedictis, Daniela Califano, Rosaria Catalano, Carmela Baviello, Silvia Varricchio, Sabrina C. Cecere, Camilla Nero, Eleonora Palluzzi, Dionyssios Katsaros, Ettore D. Capoluongo, Giovanni L. Scaglione, Sergio Marchini, Daniela Russo, Anna Spina, Laura Arenare, Francesco Morra, Maria Marotta, Marialuisa A. Vecchione, Alexandra Ingallinella, Francesco Perrone, Sandro Pignata, Angela Celetti

**Affiliations:** 1 CNR – Institute of Endotypes in Oncology, Metabolism and Immunology “G Salvatore” -IEOMI, Naples, Italy.; 2 Department of Medicine and Health Sciences, University of Molise, Campobasso, Italy.; 3 Medical Oncology Unit, University Hospital of Parma, Parma, Italy.; 4 Breast Unit, University Hospital of Parma, Parma, Italy.; 5 U.O.C Uro-Gynecology Oncology, Istituto Nazionale per lo Studio e la Cura dei Tumori-IRCCS-Fondazione “G. Pascale”, Naples, Italy.; 6 Microenvironment Molecular Targets, Istituto Nazionale per lo Studio e la Cura dei Tumori-IRCCS-Fondazione “G. Pascale”, Naples, Italy.; 7 Pathology Unit, Department of Advanced Biomedical Sciences, University of Naples Federico II, Naples, Italy.; 8 Department of Women, Children and Public Health Sciences, Fondazione Policlinico Universitario Agostino Gemelli IRCCS, Rome, Italy.; 9 Department of Surgical Sciences, Gynecology, AOU Città Della Salute, University of Torino, Turin, Italy.; 10 Department of Molecular Medicine and Medical Biotechnology, Federico II University, Naples, Italy.; 11 Bioinformatics Unit, Istituto Dermopatico dell’Immacolata, IDI-IRCCS, Rome, Italy.; 12 Cancer Pharmacology Lab, IRCCS Humanitas Research Hospital, Milan, Italy.; 13 Clinical Trial Unit, Istituto Nazionale per lo Studio e la Cura dei Tumori-IRCCS-Fondazione “G. Pascale”, Naples, Italy.

## Abstract

Patients with high-grade serous ovarian carcinoma (HGSOC) with *BRCA1/2* mutations show homologous recombination (HR) deficiency (HRD) and poly (ADP-ribose) polymerase inhibitors (PARPi) sensitivity. Notably, HRD and PARPi response can occur without *BRCA* mutations, suggesting that other factors are involved. Loss of coiled-coil domain containing 6 (CCDC6) function can lead to HRD and PARPi sensitivity in HGSOC cells, making CCDC6 a potential therapeutic target and biomarker. Three *CCDC6* missense mutations in HGSOC prompted an investigation into their impact on HRD. Analyzing CCDC6 expression, localization, and HRD data in the MITO16A trial aims to clarify the CCDC6–HRD relationship in a large cohort. The biochemical and morphologic effects of CCDC6 mutants on the native protein were examined using pull-down assays and immunofluorescence. HR-reporter and cell viability assays determined the impact of these mutants on HRD and PARPi sensitivity. The CCDC6 histochemical score and intracellular localization were assessed in MITO16A samples after immunostaining and digitalization. CCDC6-mutated isoforms act as dominant-negative, preventing native CCDC6 nuclear translocation, disrupting RAD51 foci and HR repair, and increasing PARPi sensitivity. In the MITO16A patient sample set, 66 of 185 (35%) showed barely detectable CCDC6 or nuclear exclusion (“CCDC6-inactive”). CCDC6 impairment in these “CCDC6-inactive” samples was associated with HRD in 75% (30/40) of suitable samples analyzed by the RAD51 test and in 52% (34/65) of suitable samples analyzed by genomic HRD testing, even in the presence of wild-type (WT) *BRCA1/BRCA2* genes. The association between CCDC6 inactivity and HRD, both at the genomic and functional levels, occurred even in the presence of WT *BRCA1/BRCA2* genes, suggesting that CCDC6 may play a crucial role in DNA repair pathways independent of these well-known genes.

**Significance:**

In ovarian cancer, inactivation of the CCDC6 protein signals a defect in DNA repair, known as HRD. This finding might expand the pool of patients, including those who are *BRCA* WT, who can receive PARP inhibitors, significantly broadening access to this targeted, life-extending therapy.

## Introduction

High-grade serous ovarian carcinoma (HGSOC) represents the most common and aggressive histotype of ovarian cancer, making it one of the leading causes of death from gynecologic malignancies (1). Tumors carrying *BRCA1/BRCA2* mutations exhibit the BRCAness phenotype, which is characterized by a deficiency in DNA double-strand break (DSB) repair via homologous recombination (HR; ref. 2).

This HR deficiency (HRD) can be therapeutically exploited through a synthetic lethal strategy involving the use of poly (ADP-ribose) polymerase inhibitors (PARPi; refs. 3, 4). Although initially targeting BRCA-mutated cancers, PARPi notably prolongs progression-free survival (PFS) in 50% to 60% of patients with *BRCA1/BRCA2* wild-type (WT) HGSOC, suggesting other underlying HR repair deficiencies are at play (5–7).

The critical role of HRD in predicting PARPi efficacy was cemented in 2020 when LYNPARZA gained FDA/European Medicines Agency approval for maintenance treatment in HRD-positive ovarian cancer (8, 9). Consequently, European guidelines now recommend HRD testing for all patients with advanced ovarian cancer, extending beyond just BRCA status (9–12).

Various HRD assays exist, and the RAD51 foci assessment in tumors is a promising method for predicting PARPi response, as it directly reflects HR efficiency (13–15). Low RAD51 scores correlate with PARPi sensitivity across breast, prostate, and HGSOC cancers (16–18).

Further research into other HR-related molecular events and genes is essential to improve precision medicine and expand the range of patients who can benefit from PARPi therapy.

One such protein is coiled-coil domain containing 6 (CCDC6). In various cancers, this tumor suppressor protein often loses its function due to genetic alterations, including translocations, somatic mutations, and changes in protein levels (19). Following DNA damage, CCDC6 moves to the nucleus, in which it contributes to HR repair by regulating H2AX phosphorylation through its interaction with the PP4c protein phosphatase (20).

We have recently shown that the loss of CCDC6 in HGSOC cells disrupts HR repair and induces sensitivity to PARPis, suggesting CCDC6 as a potential biomarker for PARPi response (21).

Notably, *CCDC6* is mutated in about 3% of ovarian cancer cases [Catalogue of Somatic Mutations in Cancer (COSMIC)], with three specific missense mutations (L217P, A226S, and P442S) identified at a low percentage in HGSOC. Our study characterized how these mutated isoforms of the CCDC6 protein alter the location of the native protein within the cell and how this affects its role in HR repair. A key finding was that when CCDC6 is excluded from the nucleus, its function in HR repair is compromised. This impairment of HR repair due to CCDC6 nuclear exclusion subsequently leads to increased sensitivity to PARPi in HGSOC cells. To validate these findings, we analyzed 185 samples from patients with HGSOC [formalin-fixed, paraffin-embedded (FFPE) tissues] from the MITO16A trial, which had already undergone genomic and functional HRD testing (22, 23). This analysis demonstrated a correlation between loss of CCDC6 (either due to low expression or mislocalization) and HRD. This association with HRD was particularly strong when HRD was assessed using the RAD51 assay, suggesting this functional assay may be especially sensitive to CCDC6-related HR impairment.

Given the current limitations in accurately assessing HRD, combining CCDC6 immunostaining with both genomic and functional HRD tests offers a promising approach to improve the prediction of PARPi treatment response in HGSOC. This combined strategy could be a valuable tool for identifying HRD-positive patients who are likely to benefit from PARPi therapy, and importantly, it may broaden our understanding of HRD beyond *BRCA1* and *BRCA2* mutations.

## Materials and Methods

### Cell lines, drugs, and chemicals

All the cells used in the study were authenticated using short tandem repeat profiling and were routinely checked for mycoplasma contamination using the Mycoplasma PCR Detection Kit (ABM), according to the manufacturer’s protocol. HeLa Kyoto cells (RRID: CVCL_1922), harboring the S-tag-GFP-CCDC6 construct, were generated by Ina Poser in the laboratory of Anthony Hyman and were grown in DMEM (high glucose) with 1% FCS and 1% penicillin/streptomycin (Gibco; ref. 24). Ovarian cancer cell lines OVCAR3 (RRID: CVCL_0465) and OV90 (RRID: CVCL_3768) were purchased from the ATCC and maintained in RPMI and DMEM, respectively, with 10% FBS, 1% L-glutamine, and 1% penicillin/streptomycin (Gibco). Drugs and chemicals include olaparib (AZD2281, S1060; SelleckChem), cisplatin (P4394; Merck Millipore), and Hoechst-33258 (94403; Sigma-Aldrich).

### Plasmids and transfection

pcDNA4ToA/myc-his-CCDC6 plasmid (V1030-20, Invitrogen Corporation) was transfected using FuGENE HD (Promega). Myc-CCDC6 mutants (A226S, L217P, and P442S) were generated from the WT pcDNA4ToA/myc-his-CCDC6 template using the QuickChange Site-Directed Mutagenesis Kit (Agilent). CCDC6 shRNA (pLKO.1 puro, RRID: Addgene_8453) was purchased from Sigma-Aldrich. The generation of the stable silenced cell lines (OVCAR3 and OV90) has been described elsewhere (21). Briefly, for the production of stable silenced cells, OVCAR-3 and OV90 were transfected with ShCTRL or with the ShCCDC6 plasmid pool by electroporation using the Microporator MP-100 (Digital Bio) and selected with 1 μg/mL of puromycin. The cellular population was obtained by pooling individual clones. The pDRGFP reporter plasmid (RRID: Addgene_46085) and the pCAGGS-I-SceI (RRID: Addgene_26477) have been utilized for the HR transient assay, as reported below.

### Reagents and antibodies

Antibodies against CCDC6 (Abcam cat. #ab56353, RRID: AB_940832, and Atlas Antibodies, cat. #HPA019051, RRID: AB_1846164), Rad51 (Santa Cruz Biotechnology, cat. #sc-8349, RRID: AB_2253533, and Abcam, cat. #ab133534, RRID: AB_2722613), Myc (Santa Cruz Biotechnology, cat. #sc-40, RRID: AB_627268), GFP (Takara Bio, cat. #632376, BD Living Colors), HA (Santa Cruz Biotechnology, cat. #sc-805, RRID: AB_631618), γH2AX (Merck Millipore, cat. #05-636, RRID: AB_309864), and tubulin (Sigma-Aldrich, cat. #T6557, RRID: AB_477584) were utilized for biochemical analysis and IHC. Bio-Rad provided anti-mouse and anti-rabbit secondary antibodies (cat. #170-5047, RRID: AB_11125753; cat. #170-5046, RRID: AB_11125757) for Western blot analysis. Abcam provided fluorescent anti-mouse and anti-rabbit secondary antibodies (cat. #ab150116, RRID: AB_2650601; cat. #ab150113, RRID: AB_2576208; cat. #ab150077, RRID: AB_2630356).

### Protein extraction and Western blot analysis

Total protein extracts and Western blotting procedures have been previously described (21).

### Immunofluorescence staining

Following genotoxic stress exposure [etoposide (10 μmol/L)] in transfected and control cells, immunofluorescence has been performed, as reported (25). Cells with ≥5 distinct γH2AX or RAD51 foci were considered positive. The percentage of positive nuclei was calculated from a minimum of 250 analyzed nuclei per sample.

### HR transient assay

OVCAR3 and OV90 cells transfected with myc-tagged CCDC6 mutants (A226S, L217P, P442S) or empty vector (EV) were plated in 12-well plates and transfected with the pDR-GFP reporter alone (negative control) or with pCAGGS-I-SceI. After 48 hours, cells were collected and analyzed by FACS using a MACSQuant Analyzer 10 Flow Cytometer (Miltenyi Biotec).

### Sensitivity test and design for drug combination

The CellTiter 96 AQueous One Solution Cell Proliferation Assay (Promega) was used to determine drug IC_50_ values, as described (21). CompuSyn software was used to analyze drug combinations, calculating the combination index (CI): CI = 1 (additivity), CI > 1 (antagonism), and CI < 1 (synergism).

### IHC analysis

A total of 185 HGSOC tissue samples from the MITO16A trial (NCT01706120; National Cancer Institute, Naples) were processed for IHC as described before (23). Five-micrometer sections of FFPE tissues were stained with hematoxylin and eosin or anti-CCDC6 antibody (HPA-019051, Sigma-Aldrich). CCDC6 immunoreactivity (staining intensity, fraction of stained cells, subcellular localization) was visually annotated. Digital analysis of scanned IHC slides (Leica, AT2, 40×) using QuPath quantified CCDC6 expression via a validated pipeline (watershed algorithm for cell detection, DAB intensity thresholds for classification into 0, 1+, 2+, 3+ tiers) to calculate an H-score to quantify CCDC6 expression levels (23). The median H-score across all cases was 211, and the mean was 204. Subcellular localization was also annotated. QuPath (RRID:SCR_018257) pipeline details are available upon request.

Based on the H-score and the subcellular localization of the protein, cases were categorized into inactive (defined by either an H-score <173 or complete nuclear exclusion of CCDC6) or functional (H-score >173 with diffuse or predominantly nuclear staining).

Supplementary Figure S1A presents sample size (*N*), range (min–max), average, and median H-scores for functional and inactive categories. Quartile distribution is shown in Supplementary Fig. S1B. Supplementary Figure S1C displays a flowchart summarizing the distribution of CCDC6 by functional status (active/inactive) in 122 MITO16A samples subjected to functional HRD testing. The IHC analysis of CCDC6 expression (H-score >173) and localization (diffuse or mainly nuclear) of the “CCDC6 functional” MITO16A patient set (*N* = 117) is shown in the representative images of Supplementary Fig. S2. A description of the MITO16A trial is provided in the Supplementary Methods section.

### Statistical analysis

Statistical analysis was performed using GraphPad Prism version 10 software (RRID: SCR_002798). All data are presented as mean ± SEM. One- or two-way ANOVA was used for comparative analysis between data groups. The following asterisk rating system for *P* values was used: *, *P* < 0.05; **, *P* < 0.01; ***, *P* < 0.001; ****, *P* < 0.0001.

## Results

### CCDC6 mutated isoforms interact with CCDC6 WT protein, inducing its nuclear exclusion

**Figure 1.**
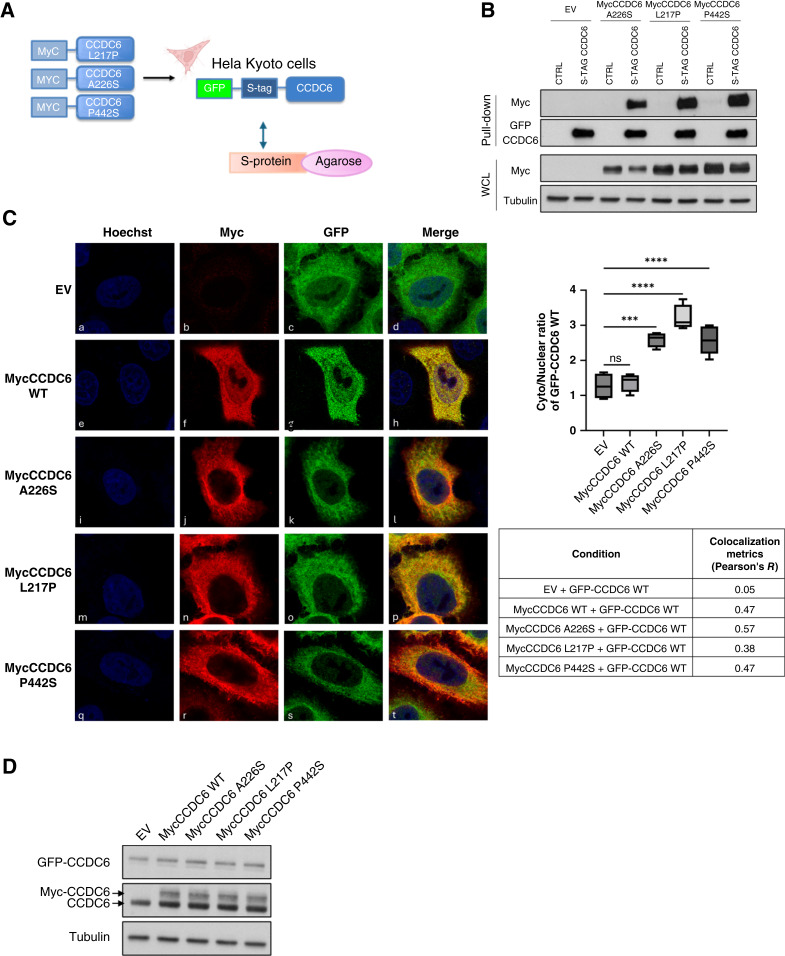
CCDC6-mutated isoforms form heterodimers with the CCDC6 WT protein and affect its intracellular distribution. **A,** List of myc-tagged CCDC6 mutants identified to date in HGSOC; schematic representation of the GFP-S-tag-CCDC6 WT construct and the S-protein agarose resin. **B,** S-tag pull-down of HeLa-Kyoto GFP-S-tag-CCDC6 and HeLa-Kyoto control cells, transfected with the myc-tagged CCDC6 mutants A226S, L217P, and P442S expression vectors or with the EV. Isolated proteins were immunoblotted with anti-myc and anti-GFP antibodies. The immunoblots of the whole cell lysates (WCL) with anti-myc, for transfection control, and anti-tubulin, as loading control, are shown at the bottom of the panel. **C,** Immunofluorescence images of HeLa-Kyoto GFP-S-tag-CCDC6 transfected with the myc-CCDC6 WT (e–h); the mutated isoforms A226S, L217P, and P442S expression vectors (i–t); or the EV as control (a–d). Nuclei are stained with Hoechst (blue channel). CCDC6 WT is shown as the endogenous GFP-S-tag-CCDC6 (green channel). The mutated isoforms were visualized by the anti-myc antibody (red channel). Quantification of the cytoplasmic-to-nuclear (Cyto/Nuclear) ratio of GFP-CCDC6 WT is shown on the right. Statistical significance was determined using one-way ANOVA (ns, not significant; ***, *P* < 0.001; ****, *P* < 0.0001). Colocalization metrics were computed using Pearson’s correlation coefficient (R) (values are reported in the table). **D,** The expression levels of CCDC6 (endogenous, GFP-tagged, and Myc-tagged forms) were assessed by Western blot using the anti-CCDC6 antibody. Tubulin is shown as a loading control.

*CCDC6*, a gene frequently mutated in cancer (COSMIC), shows 877 alterations across tumors, with missense substitutions being the most common (https://cancer.sanger.ac.uk/cosmic). Around 3% of ovarian carcinomas in COSMIC have *CCDC6* mutations. We focused on three missense mutations (L217P, A226S, P442S) in patients with HGSOC, which, despite their location, could affect CCDC6 function and localization, similar to what is seen in non–small cell lung cancer (24). To check if CCDC6 mutants interact with the normal CCDC6 protein, we introduced myc-tagged mutant versions into HeLa cells expressing GFP-S-tag-CCDC6 (WT) and control HeLa cells. Using S-protein resin to pull down the S-tagged WT CCDC6, we found that all tested myc-tagged CCDC6 mutants (L217P, A226S, P442S) could interact with the native protein (Fig. 1A and B). Confocal microscopy demonstrated that the GFP-CCDC6 WT protein showed both nuclear and cytosolic localization [Fig. 1C (c)], whereas the myc-tagged mutants were able to relocate the GFP-CCDC6 WT protein predominantly to the cytosol [Fig. 1C (k, o, s)]. A myc-tagged CCDC6 WT control was included to confirm that the impaired nuclear translocation was due to the mutations and not an artifact of the myc tag itself [Fig. 1C (g)]. These localization patterns were further validated by the merged images [Fig. 1C (d, h, l, p, t)]. This indicates that the CCDC6 mutants can heterodimerize with the WT protein and prevent its nuclear translocation. Thus, these CCDC6 mutants exhibit a dominant-negative effect on WT CCDC6 localization, causing it to be primarily cytosolic. To ensure these effects were not due to unequal protein expression, Western blot analysis was performed (Fig. 1D).

### CCDC6 mutated isoforms reduce γH2AX and RAD51 foci formation, impairing DNA repair by the HR pathway

When DNA damage occurs, CCDC6 is phosphorylated by ATM, moves to the nucleus, and aids HR repair by regulating H2AX phosphorylation through its interaction with PP4c (20, 25). As CCDC6 depletion impairs γH2AX foci formation and HR-mediated DSB repair in HGSOC cells (21, 26), we investigated the impact of CCDC6 mutants on the DNA damage response in *BRCA1/2* WT HGSOC cell lines (OVCAR3 and OV90). Immunofluorescence analysis showed that overexpressing CCDC6 mutants significantly reduced γH2AX foci formation compared with controls after treatment with genotoxic stress (Supplementary Fig. S3A and S3B). This suggests that the mutants’ inability to translocate to the nucleus disrupts DNA damage signaling. Interestingly, both conditions showed decreased RAD51 protein levels in Western blot analysis (Supplementary Fig. S3C and S3D), suggesting CCDC6 is likely involved in the HR pathway.

**Figure 2.**
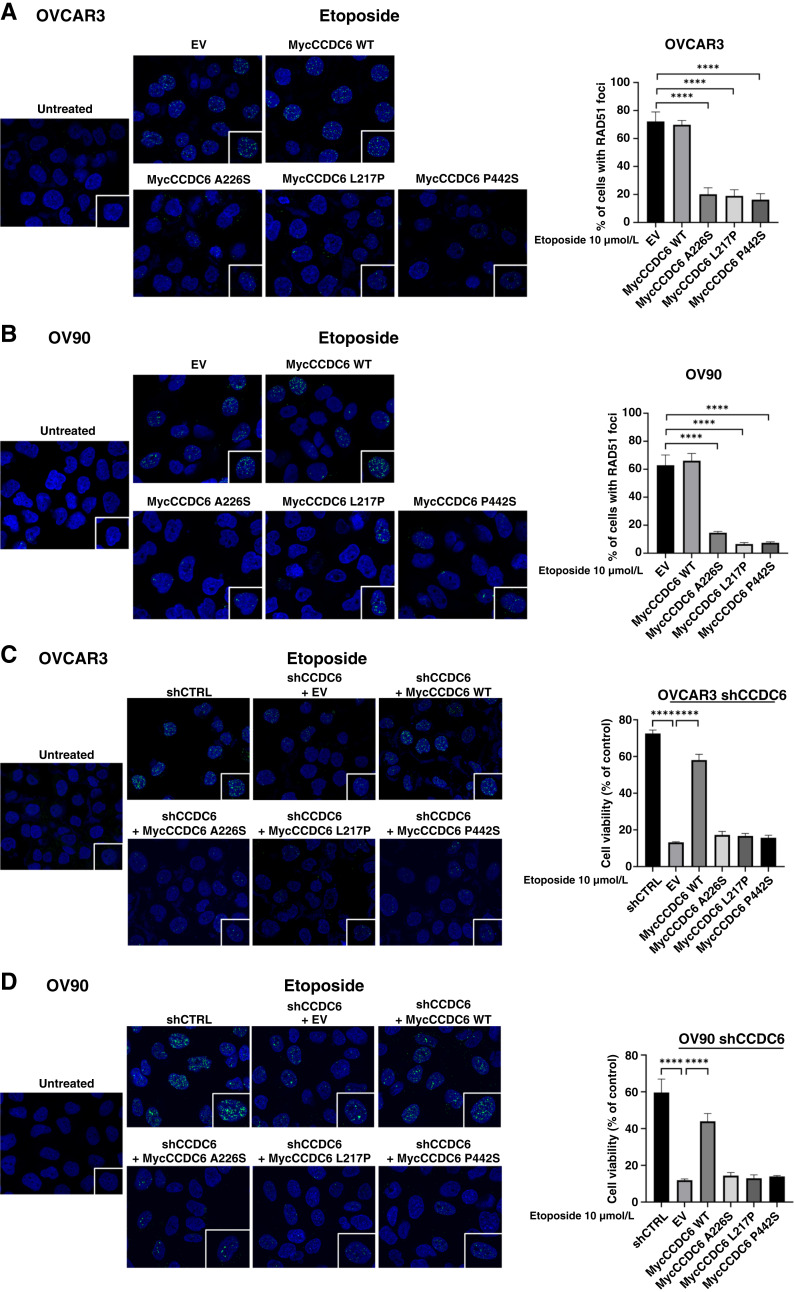
CCDC6 inactivation reduces RAD51 foci formation in HGSOC cells. **A** and **B,** Representative immunofluorescence images and quantitative analysis of RAD51 foci formation in OVCAR3 (**A**) and OV90 (**B**) cells transfected with the myc-tagged CCDC6 WT; the mutated isoforms A226S, L217P, and P442S expression vectors; or the EV as a control, following treatment with etoposide (10 μmol/L) for 8 hours. Graphs represent the percentage of cells with more than five foci. Error bars indicate the SEM derived from three independent experiments. Statistical significance was verified by one-way ANOVA (****, *P* < 0.0001). **C** and **D,** Representative immunofluorescence images and quantitative analysis of RAD51 foci formation in OVCAR3 (**C**) and OV90 (**D**) cells stably silenced for CCDC6 (shCCDC6) and transfected with the myc-tagged CCDC6 WT; the mutated isoforms A226S, L217P, and P442S expression vectors; or the EV and control cells (shCTRL), following treatment with etoposide (10 μmol/L) for 8 hours. Graphs represent the percentage of cells with more than five foci. Error bars indicate the SEM derived from three independent experiments. Statistical significance was verified by one-way ANOVA (****, *P* < 0.0001).

RAD51 is essential for accurate HR repair at DNA breaks. After damage, RAD51 forms nuclear foci at these sites, indicating active HR (27). To understand CCDC6’s role in HR, we examined RAD51 foci formation in *BRCA1/2* WT HGSOC cells with either CCDC6 mutant overexpression or stable CCDC6 depletion. Both CCDC6 loss and mutant overexpression led to reduced RAD51 recruitment to nuclear foci compared with controls (Fig. 2A–D; Supplementary Fig. S4). A WT CCDC6 control has been included in Fig. 2 to clarify that the observed effects are mutation-specific, ruling out artifacts from the expression system or protein overexpression. To specifically demonstrate that CCDC6’s nuclear versus cytoplasmic localization affects HR in a CCDC6-null HGSOC background, we transiently transfected CCDC6-depleted HGSOC cells and control cells with myc or GFP EVs, CCDC6WT, and the CCDC6 point mutant constructs. As detailed in Supplementary Fig. S5, we demonstrate that the CCDC6 mutants exhibit a predominantly cytosolic localization in the CCDC6-null HGSOC background, mirroring the results observed in HeLa cells (Fig. 1). Furthermore, the impaired nuclear localization of these mutants in the CCDC6-null HGSOC background directly correlates with a significantly decreased HR activity, as evidenced by reduced Rad51 foci formation (Fig. 2C and D). These data confirm that the nuclear versus cytoplasmic localization of CCDC6 specifically affects HR in HGSOC cells.

Finally, to assess whether CCDC6 mutants affect HR repair efficiency, we expressed myc-tagged L217P, A226S, and P442S mutants with the DR-GFP reporter and HA-I-SceI endonuclease in OVCAR3 and OV90 cells. After 48 hours, flow cytometry showed fewer fluorescent (recombined) cells in those expressing each CCDC6 mutant compared with control cells (Supplementary Fig. S3E and S3F).

These findings indicate that CCDC6 mutants, similar to CCDC6 depletion (21), impair HR repair in HGSOC cells.

### The CCDC6 mutated isoforms determine PARPi and cisplatin sensitivity in HGSOC cells

**Figure 3.**
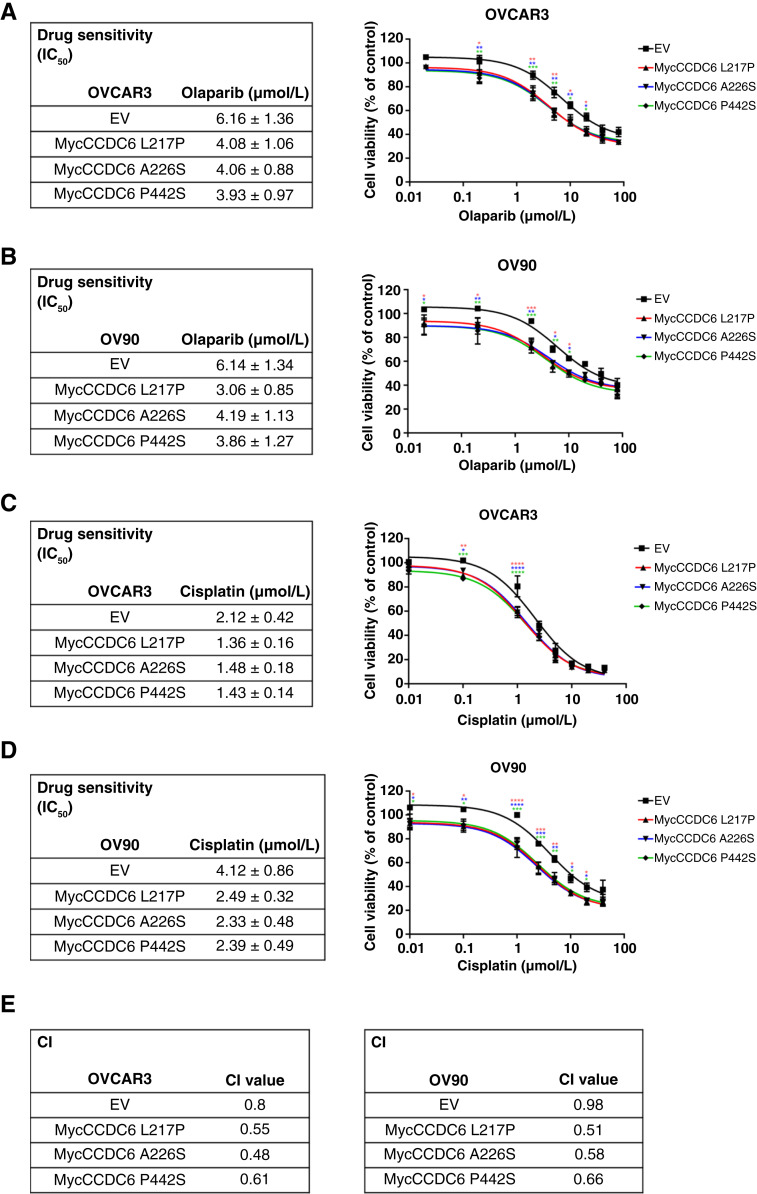
Mutated isoforms of CCDC6 sensitize HGSOC cells to the PARPi olaparib alone or in combination with cisplatin. **A–D,** Drug sensitivity to olaparib or cisplatin has been evaluated by cell viability assay (CellTiter 96 Aqueous One Solution assay, Promega) in OVCAR3 and OV90 cell lines transfected with the myc-CCDC6 mutated isoforms L217P, A226S, and P442S or with the EV and exposed to the drug for 144 hours. The drug sensitivity is expressed as IC_50_ values. Statistical significance was verified by two-way ANOVA (*, *P* < 0.05; **, *P* < 0.01; ***, *P* < 0.001; ****, *P* < 0.0001). **E,** The CI values according to the 1:2 concentration ratio of cisplatin and olaparib are shown (CI < 1, CI = 1, and CI > 1 indicate synergism, additive effect, and antagonism, respectively).

HR deficiency predicts PARPi sensitivity and guides treatment decisions (4, 28). We investigated whether CCDC6 dysfunction, due to its exclusion from the nucleus, could lead to PARPi sensitivity in HGSOC cells. Expressing CCDC6 mutants in OVCAR3 and OV90 cells increased their sensitivity to olaparib (Fig. 3A and B) and cisplatin (Fig. 3C and D). Notably, CCDC6 mutant overexpression further enhanced the synergy between olaparib and cisplatin (Fig. 3E). Our findings suggest that in HGSOC cells, the loss of WT CCDC6 nuclear activity in HR creates a synthetic lethal vulnerability to PARP inhibition, contributing to the observed drug synergy.

### The physical or functional loss of CCDC6 confers an HRD phenotype in patients with HGSOC from MITO16A

We sought to establish a link between CCDC6 loss (physical or functional) and HRD in patients with HGSOC. For this analysis, we used 185 FFPE tissue samples from the MITO16A clinical trial (23, 29). We evaluated CCDC6 protein expression and localization in these samples using IHC and digital image analysis (QuPath). CCDC6 protein levels were quantified using an H-score, which integrates both staining intensity and the percentage of positive cells. Besides total protein levels, we analyzed CCDC6’s subcellular localization (nucleus vs. cytosol) to see if its distribution correlates with HRD status.

**Figure 4.**
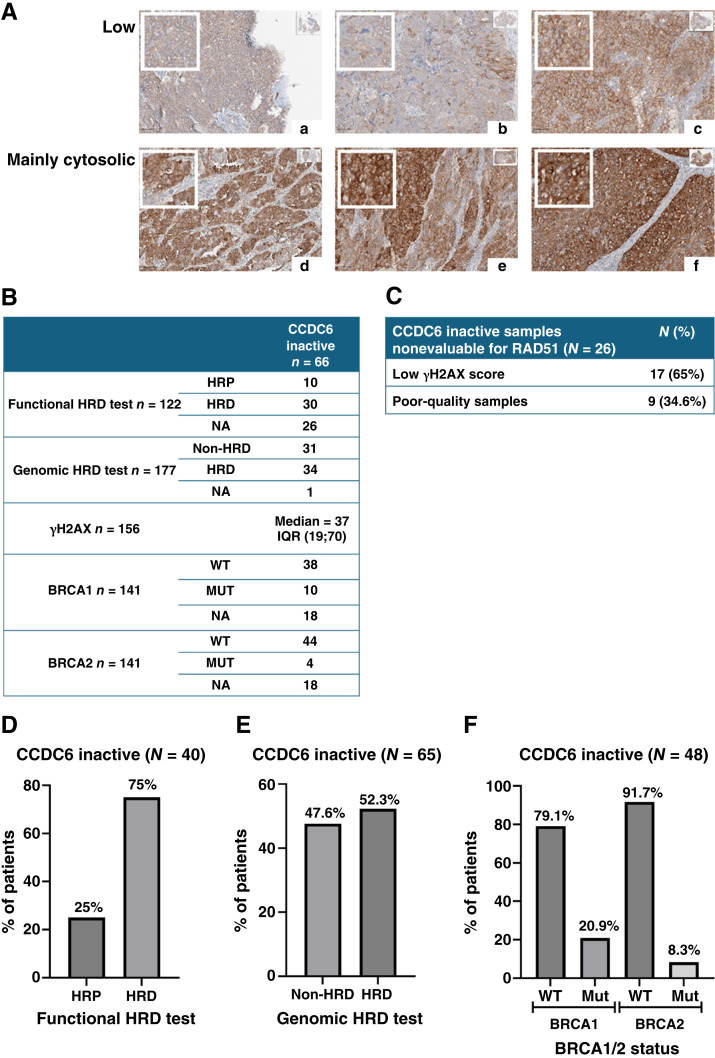
CCDC6 inactivation correlates with an HRD phenotype in patients with HGSOC. **A,** Representative images of IHC analysis of CCDC6 expression (a–c) and localization (d–f) in FFPE MITO16A patient samples (magnification 40×). The H-score values are listed as follows: a, (9); b, (50.68); c, (138.86); d, (149.61); e, (194.51); and f, (262.82). **B,** Summary of the MITO16A cases with CCDC6 inactivation, assessed for HRD test and/or RAD51 test and/or *BRCA1/2* status. **C,** Causes of failure for the RAD51 assay in CCDC6 inactive samples. **D,** Percentages of Homologous Recombination Proficiency (HRP) and HRD tumors assessed by functional HRD testing among CCDC6 inactive MITO16A samples. **E,** Percentages of non-HRD and HRD tumors assessed by genomic HRD testing among CCDC6 inactive MITO16A samples. **F,** Percentage of *BRCA1* and *BRCA2* WT or mutated tumors among CCDC6-inactive MITO16A samples.

In the MITO16A cohort, 66 of 185 samples (35%) were classified as “CCDC6 inactive” due to either complete nuclear exclusion or very low expression (H-score <173; Fig. 4A).

The MITO16A trial samples had previously been analyzed for HRD using both genomic and functional (RAD51 foci) tests, and BRCA1/2 mutational status was also available, as summarized in Fig. 4B.

The innovative RAD51 immunofluorescence assay was used in the MITO16A trial to functionally assess HR repair capability in a subset of suitable patient samples (122 of 185 patients with HGSOC; Supplementary Fig. S1C; refs. 18, 22, 23). Seventy-five percent (30/40) of the “CCDC6-inactive” samples were found to be HRD-positive by this functional assay, providing strong support for our *in vitro* findings (Fig. 4D). Genomic testing showed that 52% (34/65) of CCDC6-inactive samples also exhibited genomic HR deficiency (Fig. 4E). The slightly lower percentage compared with the functional assay may be due to limitations in the genomic test (18, 23, 29).

Notably, the “CCDC6 inactive” group was predominantly found among *BRCA1* and *BRCA2* WT patients (Fig. 4F), suggesting that CCDC6 impairment may be mutually exclusive with *BRCA1/2* mutations.

Of the 26 “CCDC6-inactive” samples that lacked a RAD51 assay result, 17 (65%) showed a low γH2AX score (<25% of geminin-positive cells with γH2AX foci; Fig. 4C). This further indicates CCDC6 impairment and suggests these patients also have HRD. Our analysis confirms that “CCDC6 inactive” status, reflecting physical or functional CCDC6 loss, is strongly associated with an HRD phenotype.

This finding is clinically significant because assessing CCDC6 status could identify patients with HRD who might be missed by current RAD51 assays. This suggests that PARPi therapy may benefit a broader group of patients beyond those with established *BRCA* mutations. Further research is required to validate this expanded clinical utility.

## Discussion

Patients with HGSOC with *BRCA1/2* mutations exhibit HRD, leading to sensitivity to PARPis. However, HRD and PARPi sensitivity can occur independently of *BRCA* mutations (1–4). This study identifies the loss of CCDC6 function as a novel biomarker and therapeutic target, linking CCDC6 inactivity to HRD and increased PARPi sensitivity. Functional assays and IHC analysis of MITO16A trial samples revealed that 35% of HGSOC cases exhibited CCDC6 inactivity, with a strong association with HRD by RAD51 and genomic HRD testing. These findings suggest a potential expansion of PARPi eligibility beyond *BRCA*-mutant patients.

*CCDC6* has been proposed as a tumor suppressor gene due to its role in the surveillance of DNA integrity (20, 25). Preclinical data demonstrated an association between *CCDC6* gene product depletion in HGSOC cells and olaparib sensitivity, suggesting CCDC6 as a potential biomarker of the BRCAness phenotype and PARPi sensitivity (21).

The easier accessibility of NGS technology and advancements in diagnostic tools have led to a significant increase in omics data, including extensive information on *CCDC6* molecular alterations in online databases. These alterations are highly heterogeneous, involving fusions with various partners and point mutations clustered in different coding regions. A systematic characterization of *CCDC6* alterations in HGSOC was lacking, and we questioned whether these could cause a CCDC6 loss-of-function phenotype.

The characterized *CCDC6* missense mutations, found heterozygously in HGSOC, produce a phenotypic effect similar to that of native protein depletion. Furthermore, our data elucidate the mechanism, as overexpressed mutant CCDC6 isoforms cause nuclear exclusion of the WT protein, leading to a CCDC6 loss of function not explained by the Knudson two-hit theory. This dominant-negative behavior has been previously observed with truncated and mutated CCDC6 isoforms in other cancer cell models (24).

The CCDC6 mutants A226S, L217P, and P442S retain the ability to heterodimerize with WT CCDC6, causing altered subcellular localization and impaired native protein function. Immunofluorescence showed reduced nuclear localization of WT CCDC6 with these mutants, indicating that cytosolic delocalization/nuclear exclusion disrupts the CCDC6–PP4c interaction (20, 21). This disruption leads to unrestrained PP4c activity, affecting H2AX phosphorylation, DNA damage signaling, cell-cycle progression, and HR-mediated DSB repair, as evidenced by a decrease in GFP-positive cells in the HR DR-GFP reporter assay upon mutant expression.

Given that CCDC6 mutants primarily delocalize the native protein to the cytosol, we emphasized the importance of evaluating CCDC6 intracellular localization (cytosol/nucleus), in conjunction with CCDC6 protein intensity, in tissue samples from patients with HGSOC. This assessment was performed via IHC on a large cohort of HGSOC specimens from the MITO16A clinical study (23). We classified 66 out of 185 analyzed samples (35%) as “CCDC6 inactive,” based on either complete nuclear exclusion or an H-score within the first quartile (H-score <173), signifying null or minimal CCDC6 expression (Supplementary Fig. S1A).

Consistent with our *in vitro* observation that CCDC6 loss or nuclear exclusion reduces RAD51- and γH2AX-positive cells and impairs HR repair, MITO16A patients with “CCDC6-inactive” tumors predominantly showed HR impairment, as confirmed by genomic HRD testing and, notably, the RAD51 functional assay.

However, the RAD51 assay had a 30% failure rate due to sample issues or low DNA damage (i.e., low γH2AX score; ref. 23). Considering that CCDC6 physical or functional impairment significantly reduces γH2AX foci formation, we conclude that “CCDC6 inactive” status seems to be a valuable tool to identify patients with HRD eligible for PARPi therapy who might otherwise be excluded based solely on RAD51 assay unsuitability. Additional investigation is necessary to reinforce this observation.

Overall, the CCDC6 mutated isoforms, behaving in a dominant negative manner versus the CCDC6 native protein, confer to the tumoral cells an increased genomic instability due to the defective HR and a vulnerability exploitable by precision medicine approaches, as shown *in vitro* by enhanced sensitivity to PARPi treatment and verified by cell viability assays. The enhanced cytotoxic effect of olaparib and the increased synergistic effect of PARPi when combined with other DNA-damaging agents, such as cisplatin, support a BRCAness phenotype dependent on CCDC6 mutated isoforms that we have characterized.

Here, we have learned that DNA mutations can mislocalize CCDC6, impairing its nuclear function in HGSOC. Future work should explore other mechanisms, including CCDC6 interaction with nuclear import/export proteins like XPO1 (30). Changes in CCDC6 posttranslational modification sites (by kinases, phosphatases, or stability enzymes) might also increase turnover, alter dynamics, and affect protein complex interactions (20, 25). Furthermore, CCDC6’s role in DDR and HR is not fully understood. Computational analysis suggests that CCDC6 interacts with HR proteins like BRCC3 and BAP1 (of the BRCA1 complex; ref. 31), and ongoing work explores a potential interaction with PALB2. Future studies will clarify the biochemical dynamics of CCDC6 interactions (native and mislocalized forms) under various conditions. Beyond point mutations, uncharacterized gene rearrangements, such as the *CCDC6–ANK3* fusion in HGSOC (32), also warrant investigation.

Notably, the observation that the CCDC6 mutants have the ability to mainly delocalize the native protein in the cytosol suggests, besides the intensity, a careful evaluation of the CCDC6 intracellular localization (cytosol/nucleus) in tissue samples of patients with HGSOC. In this work, this kind of evaluation has been pursued by IHC in a large series of HGSOC specimens obtained from the MITO16A clinical trial that also provided patients with information about genomic and functional HRD tests.

It is of interest that the study demonstrated a significant association between CCDC6 inactivity and HRD, as evidenced by both genomic and functional analyses. Of particular significance is the observation that this association was maintained in the presence of WT *BRCA1/BRCA2* genes, suggesting a crucial role for *CCDC6* in DNA repair pathways that are independent of these well-characterized genes.

However, the MITO16A study primarily aimed to identify clinical and biological factors predicting PFS or overall survival in patients receiving first-line chemotherapy and bevacizumab, rather than focusing on PARPi response. We are currently investigating CCDC6 behavior in *BRCA* WT patients with newly diagnosed advanced epithelial ovarian cancer who achieved a partial or complete response to first-line platinum-based chemotherapy and are enrolled in the multicenter, prospective, single-arm MITO35A trial of olaparib maintenance therapy. Thus, the MITO35a findings could translate MITO16A’s initial observations into clinical practice.

In conclusion, our findings establish CCDC6 inactivity, defined by reduced expression or nuclear exclusion, as a novel and clinically actionable biomarker for HRD in HGSOC. Importantly, CCDC6 status identifies HRD-positive tumors even in the presence of WT *BRCA1/2* genes, thereby expanding the population of patients potentially eligible for PARPi therapy. The combination of CCDC6 IHC assessment with functional RAD51 assays provides a promising strategy to refine patient selection for precision medicine approaches. Future validation in independent cohorts and prospective clinical trials will be essential to integrate CCDC6 evaluation into standard HRD diagnostic workflows, with the ultimate goal of improving therapeutic outcomes for a broader subset of patients with HGSOC.

## Supplementary Material

Figure S1 Figure S1. Summary statistics of H-scores for protein localization categories.

Figure S2 Figure S2. IHC analysis of CCDC6 staining intensity and localization; CCDC6 expression evaluated by IHC compared to negative IgG control.

Figure S3 Figure S3: (A) Immunofluorescence shows yH2AX nuclear foci formation in OVCAR3/OV90 cells after transfection with myc- CCDC6 mutated isoforms (A226S, L2 l 7P, P442S) or Empty Vector (EV) control and treatment with Etoposide [10 µM] for 8 hours. (B) Graphs display the percentage of cells with >5 foci (±SEM). (C-D) Western blots detect RAD5 l protein levels following transfection with CCDC6 mutated isoforms (C) or stable CCDC6 silencing (D) in OVCAR3/OV90 cells. Anti- Myc/Anti-CCDC6 and Anti-Tubulin were used as transfection/depletion and loading controls, respectively. (E-F) OVCAR3/OV90 cells, transfected with Myc-CCDC6 mutated isoforms/EV plus DR-GFP construct and I-Seel enzyme, were assessed for Homology-Directed Repair (HDR) efficiency (percentage of GFP positive cells, ±SEM).

Figure S4 Figure S4: (A) Levels of endogenous CCDC6, MycCCDC6 WT and mutated isoforms overexpression were assessed in OVCAR3 and OV90 cells by Western Blot using an anti-CCDC6 antibody. Tubulin is shown as loading control. (B) Levels ofMyc-CCDC6 WT and mutated isoforms overexpression and the degree of CCDC6 silencing were assessed in OVCAR3 and OV90 cells by Western Blot using an anti-CCDC6 antibody. Tubulin is shown as loading control. (C-D) Following treatment with Etoposide [10 µM] for 8 hours, cell viability was assessed in OVCAR3 and OV90 cells (C) as well as in OVCAR3 and OV90 cells stably silenced for CCDC6 (shCCDC6) (D) transfected with the myc-tagged CCDC6 wild type (WT), the mutated isoforms, A226S, L2l 7P and P442S expression vectors, or the empty vector (EV). Error bars indicate the standard error mean derived from three independent experiments.

Figure S5 Figure S5: Immunofluorescence images of OVCAR3 cells stably silenced for CCDC6 (shCCDC6) or control cells (shCTRL) transfected with the GFP vector (a-d) or the GFP-CCDC6 wild type (WT) co-transfected with the myc-tagged CCDC6 wild type (i-1), the mutated isoforms A226S, L217P and P442S expression vectors (m-x) or the empty vector (EV) as control (e-h). Nuclei are stained with Hoechst (Blue-channel). GFP-CCDC6 WT protein is shown in the green channel. The myc-tagged CCDC6 WT and mutated isoforms were visualized by the anti-myc antibody (red-channel).

Supplementary Methods Supplementary Methods

## Data Availability

The data generated in this study are available from the corresponding author upon request.
