# Supplementary figures and images for: CCDC6 Immunostaining in Conjunction with the Rad51 HRD Assay May Expand PARPi Treatment Eligibility in Patients with HGSOC
Source: Cancer Res Commun. 2026 Jan 26;6(1):201–10. doi: 10.1158/2767-9764.CRC-25-0455 (PMC12833555; doi:10.1158/2767-9764.CRC-25-0455)

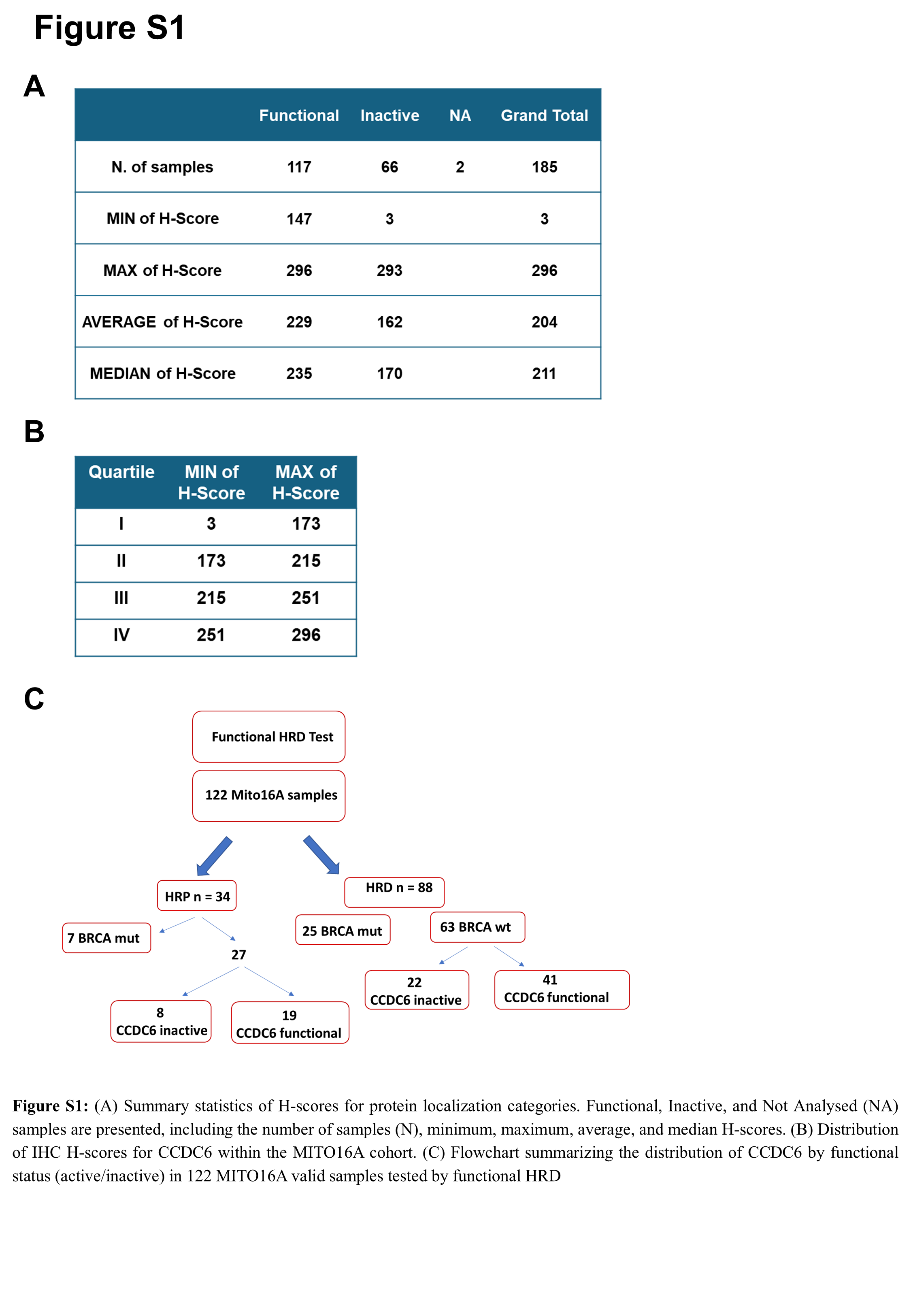

Supplement: Figure S1 — Summary statistics of H-scores for protein localization categories. [file crc-25-0455_figure_s1_suppsf1.png]

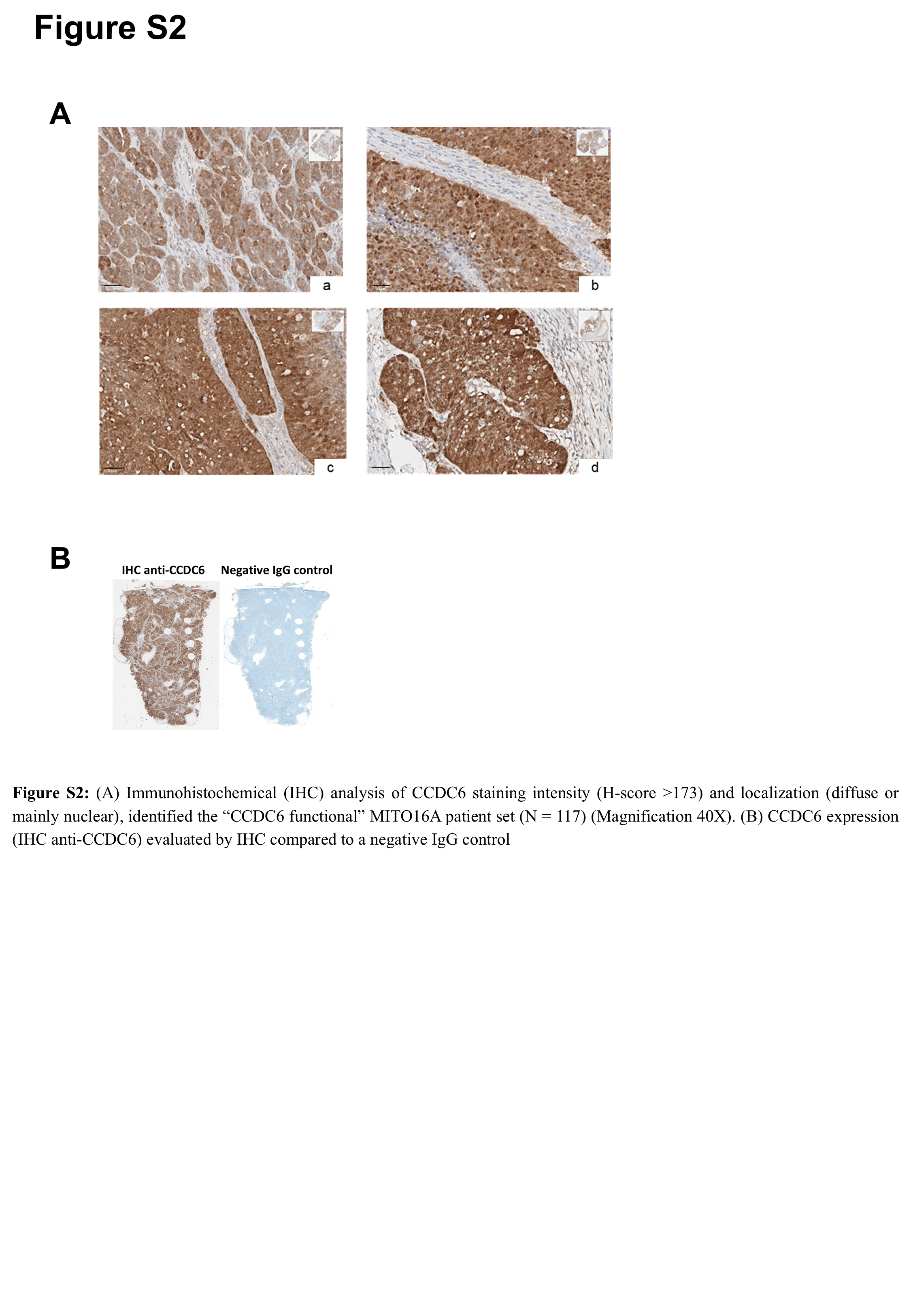

Supplement: Figure S2 — IHC analysis of CCDC6 staining intensity and localization; CCDC6 expression evaluated by IHC compared to negative IgG control. [file crc-25-0455_figure_s2_suppsf2.png]

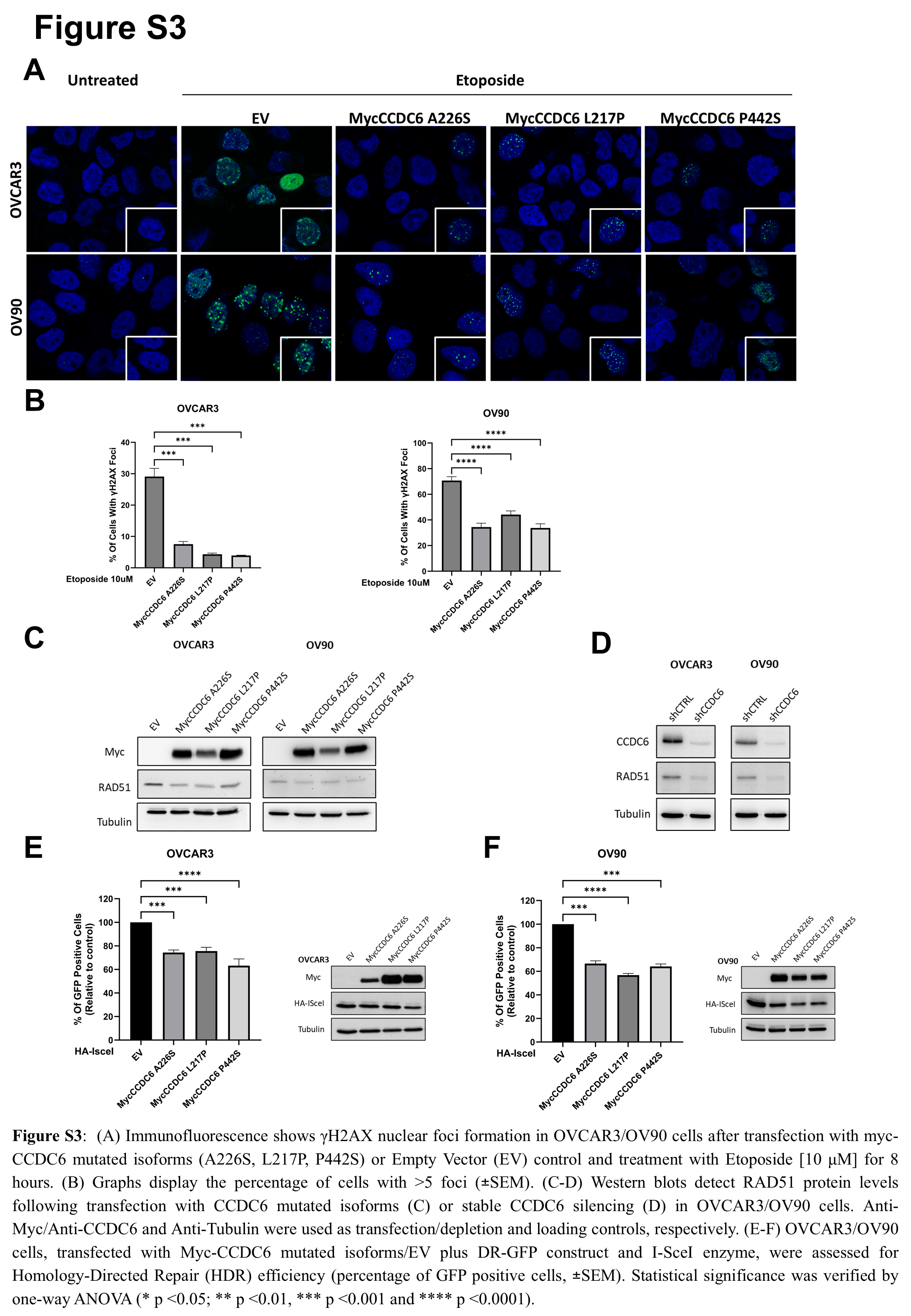

Supplement: Figure S3 — (A) Immunofluorescence shows yH2AX nuclear foci formation in OVCAR3/OV90 cells after transfection with myc- CCDC6 mutated isoforms (A226S, L2 l 7P, P442S) or Empty Vector (EV) control and treatment with Etoposide [10 µM] for 8 hours. (B) Graphs display the percentage of cells with >5 foci (±SEM). (C-D) Western blots detect RAD5 l protein levels following transfection with CCDC6 mutated isoforms (C) or stable CCDC6 silencing (D) in OVCAR3/OV90 cells. Anti- Myc/Anti-CCDC6 and Anti-Tubulin were used as transfection/depletion and loading controls, respectively. (E-F) OVCAR3/OV90 cells, transfected with Myc-CCDC6 mutated isoforms/EV plus DR-GFP construct and I-Seel enzyme, were assessed for Homology-Directed Repair (HDR) efficiency (percentage of GFP positive cells, ±SEM). [file crc-25-0455_figure_s3_suppsf3.png]

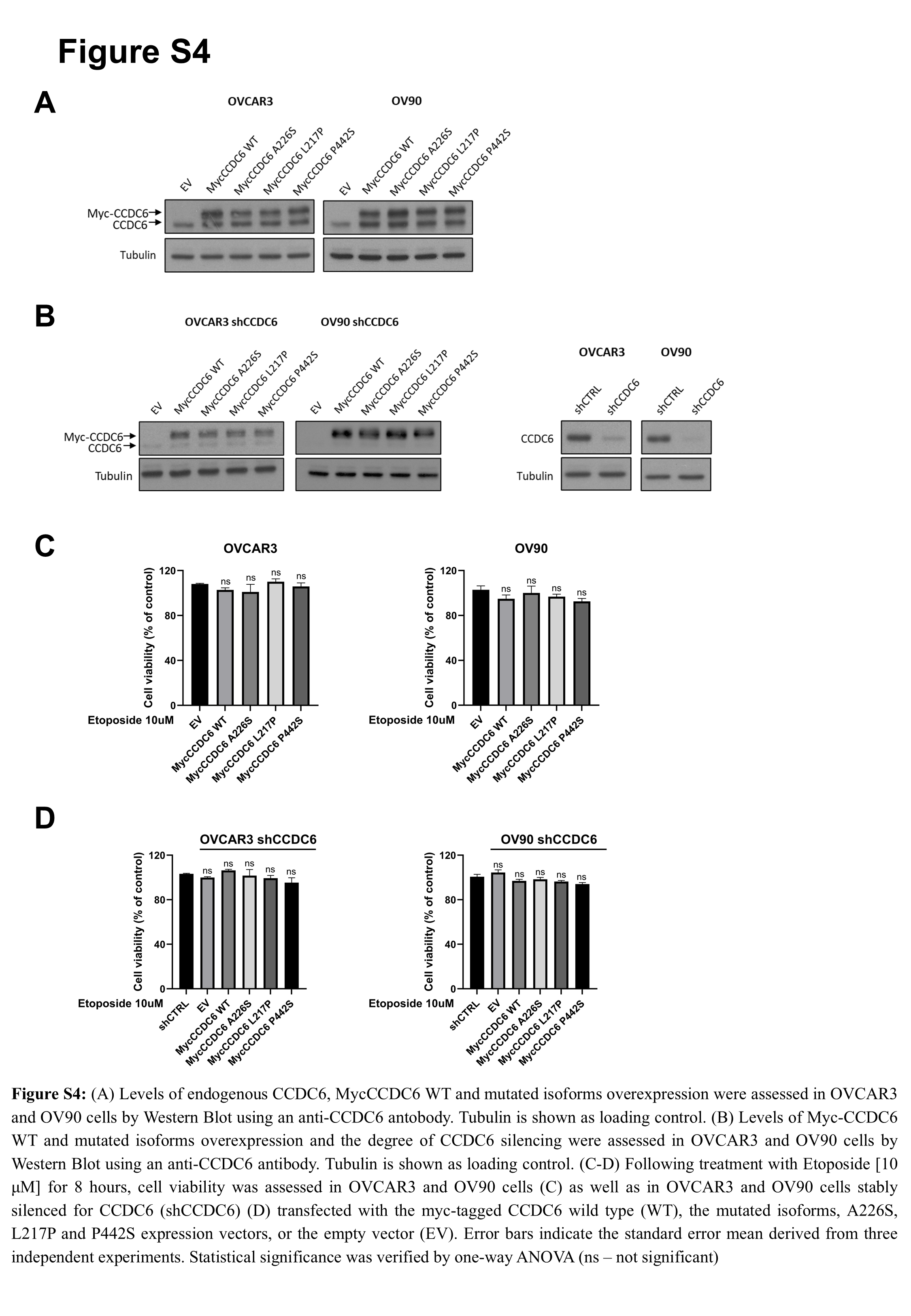

Supplement: Figure S4 — (A) Levels of endogenous CCDC6, MycCCDC6 WT and mutated isoforms overexpression were assessed in OVCAR3 and OV90 cells by Western Blot using an anti-CCDC6 antibody. Tubulin is shown as loading control. (B) Levels ofMyc-CCDC6 WT and mutated isoforms overexpression and the degree of CCDC6 silencing were assessed in OVCAR3 and OV90 cells by Western Blot using an anti-CCDC6 antibody. Tubulin is shown as loading control. (C-D) Following treatment with Etoposide [10 µM] for 8 hours, cell viability was assessed in OVCAR3 and OV90 cells (C) as well as in OVCAR3 and OV90 cells stably silenced for CCDC6 (shCCDC6) (D) transfected with the myc-tagged CCDC6 wild type (WT), the mutated isoforms, A226S, L2l 7P and P442S expression vectors, or the empty vector (EV). Error bars indicate the standard error mean derived from three independent experiments. [file crc-25-0455_figure_s4_suppsf4.png]

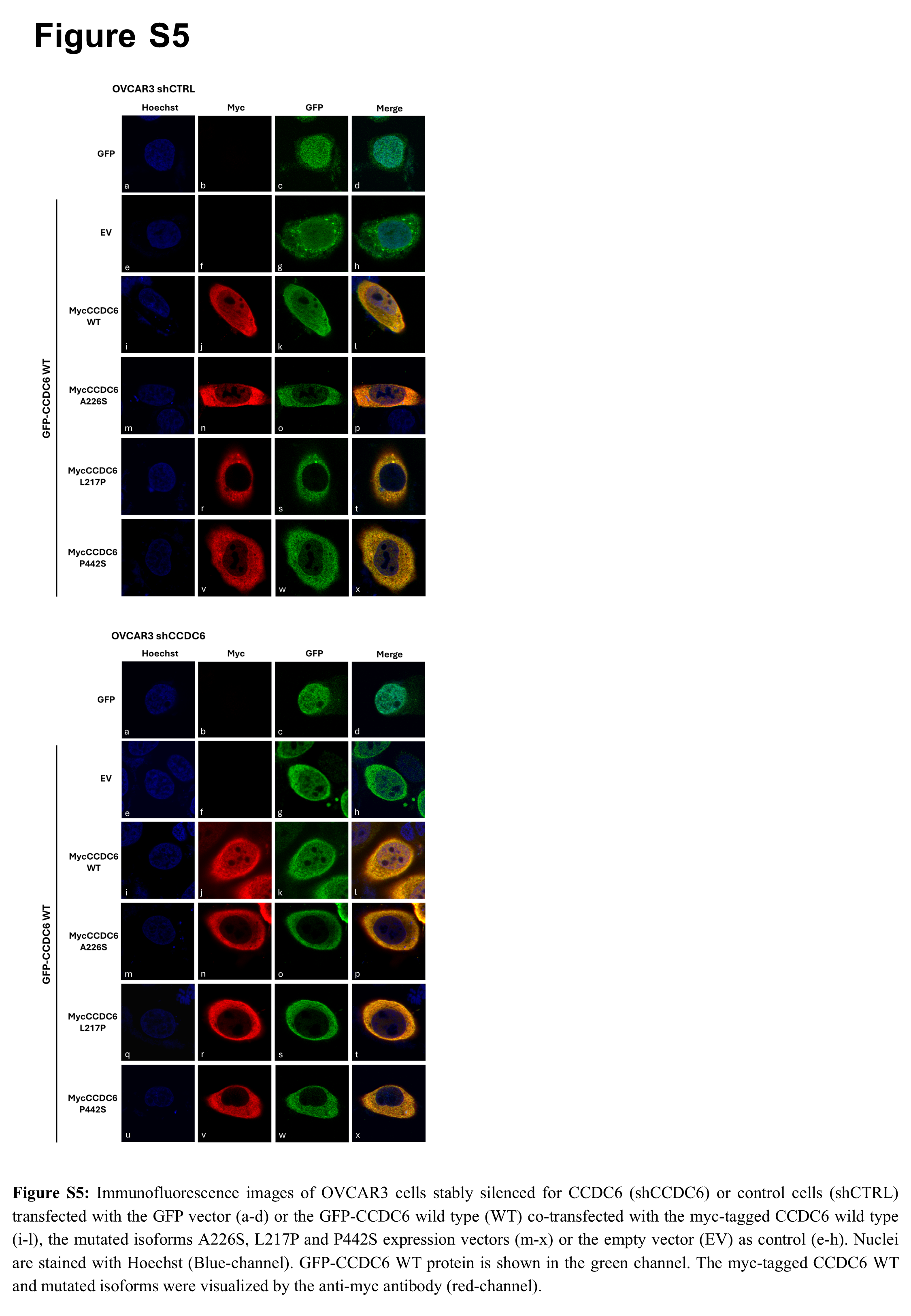

Supplement: Figure S5 — Immunofluorescence images of OVCAR3 cells stably silenced for CCDC6 (shCCDC6) or control cells (shCTRL) transfected with the GFP vector (a-d) or the GFP-CCDC6 wild type (WT) co-transfected with the myc-tagged CCDC6 wild type (i-1), the mutated isoforms A226S, L217P and P442S expression vectors (m-x) or the empty vector (EV) as control (e-h). Nuclei are stained with Hoechst (Blue-channel). GFP-CCDC6 WT protein is shown in the green channel. The myc-tagged CCDC6 WT and mutated isoforms were visualized by the anti-myc antibody (red-channel). [file crc-25-0455_figure_s5_suppsf5.png]
