# Supplementary material for: CCDC6 Immunostaining in Conjunction with the Rad51 HRD Assay May Expand PARPi Treatment Eligibility in Patients with HGSOC
Source: Cancer Res Commun. 2026 Jan 26;6(1):201–10. doi: 10.1158/2767-9764.CRC-25-0455 (PMC12833555; doi:10.1158/2767-9764.CRC-25-0455)
Supplement: Supplementary Methods [file crc-25-0455_supplementary_methods_suppsm.docx]

**Supplementary Methods:**

**Description of the MITO16A trial:**

The MITO16A/MaNGO-OV2 (www.clinicaltrials.gov number: NCT01706120 or EudraCT number: 2012-003043-29, hereafter indicated as MITO16A) is a single arm, multicenter, open-label, non-comparative, phase IV trial of first-line chemotherapy (carboplatin plus paclitaxel) and bevacizumab, followed by bevacizumab as single agent until progression or up to 22 total cycles. Overall, 398 patients were prospectively enrolled from 47 participant centers. Twelve research groups designed the trial as an exploratory study and no “a priori” hypothesis was defined to calculate the sample size of the trial. The primary aim of the MITO16A was to study clinical and biological factors for their value as prognostic factors for progression-free survival (PFS)

Formalin-fixed, paraffin-embedded (FFPE) blocks were collected and stored and dedicated standard operating procedures (SOPs) for collection, shipping, and processing were developed and made available to MITO researchers through the coordinating center’s web-based platform. The coordinating center collected FFPE blocks from each local center, with an anonymized copy of the pathology report, a hematoxylin and eosin (H&E) stained slide.

The FFPE blocks were identified with the MITO patient’s ID and stored under ambient conditions until processing. Samples were processed centrally and the materials were distributed to the participating labs for biomarker analysis.
